# Regional Nerve Block Complication Analysis Following Peripheral Nerve Block During Foot and Ankle Surgical Procedures

**DOI:** 10.7759/cureus.9434

**Published:** 2020-07-28

**Authors:** Jason A Lauf, Pearson Huggins, Joseph Long, Mohammed AL-Issa, Brian Byrne, Bryan P Large, Brent Whitehead, Nicholas A Cheney, Timothy D Law

**Affiliations:** 1 Orthopedic Surgery, Ohio University Heritage College of Osteopathic Medicine, Dublin, USA; 2 Medicine, Ohio State University College of Medicine, Columbus, USA; 3 Emergency Medicine, Mercy St. Elizabeth Boardman Hospital, Youngstown, USA; 4 Anesthesiology, OhioHealth Doctors Hospital, Columbus, USA; 5 Foot and Ankle Surgery, OrthoNeuro, Columbus, USA; 6 Family Medicine, Ohio University Heritage College of Osteopathic Medicine, Athens, USA

**Keywords:** peripheral nerve block, outcome studies, statistical analysis, popliteal block, complications, demographics

## Abstract

Background

Foot and ankle surgeries are frequently accompanied by a peripheral nerve block in order to reduce postoperative pain. Higher than expected complication rates with peripheral nerve blocks have led to increased concern among surgeons and patients. To our knowledge, no study conducted by the treating surgeon has identified risk factors that may predispose a patient to complications. Our goal was to attempt to identify those risk factors.

Methods

We reviewed patient charts of those who underwent an orthopedic foot and ankle procedure between 2013 and 2018, as performed by the senior author. This yielded 992 procedures performed across four surgical locations. Of these procedures, 137 procedures were removed because no block was used. The remaining cases were analyzed for nerve complications, defined as sensory or motor deficits along the distribution of a nerve. The patients were divided into those with and without complications and were evaluated for differences. Statistical analysis was performed using the SAS^®^ software (SAS Institute Inc., Cary, North Carolina, USA).

Results

The overall short-term complication rate was 10.1% and the long-term complication rate was 4.3%, with a total of 855 blocks given. Electromyographies (EMGs) were performed on 24.4% of the patients with a complication. Of the EMGs, 95.2% confirmed nerve complications in the distribution of the blocked nerve. The significant factors associated with complications were age, BMI, location, and smoking status. A regression analysis was performed to determine the odds ratio for individual factors. Those with significantly higher odds ratio were between 40 and 65 years of age, had normal or underweight BMI, underwent surgery at an outpatient surgery center, and were current smokers.

Conclusions

Our study suggests that there are significant epidemiological factors in predicting postoperative complications related to a peripheral nerve block. The study also shows a similar short-term complication rate but a higher long-term complication rate than other studies. This data are important because it allows for an informed decision to be made between a surgeon, anesthesiologist, and the patient regarding the safety and necessity of delivering a preoperative peripheral nerve block based on patient risk factors.

## Introduction

Postoperative pain can be a standard problem for patients undergoing foot and ankle surgery. One method of trying to help manage postoperative pain is peripheral nerve blocks. This involves an injection of local anesthetic, most commonly under ultrasound guidance, either with or without a nerve stimulator, adjacent to a peripheral nerve in order to induce paresthesia in the distribution of the nerve. Previous studies have shown higher success rates when using an ultrasound to guide peripheral nerve blocks [1-4]. Studies have also shown increased success with a combination of ultrasound guidance and nerve stimulation when compared to neurostimulation alone [2]. The most commonly targeted nerve in foot and ankle surgery is the popliteal nerve [5].

Although beneficial, there are complications that can be associated with peripheral nerve blocks. Complications can range from injection site infection to motor dysfunction [6]. Most of the current studies have been performed by anesthesiologists and have shown a low rate of nerve irritation that was attributed to the nerve block [6-12]. Studies have been conducted to assess the long-term complication rates of peripheral blocks [7-16]. These studies focused solely on the complication rates. Anderson et al. [13] expanded in the available literature by examining factors such as tourniquet time and epinephrine use in predicting the rate of complications. Gartke et al. [17] also performed a multivariable analysis in a prospective study that included both clinical and demographic factors. The exact mechanism of the residual nerve symptoms remains unknown, and multiple potential causes in addition to the peripheral nerve blocks have been implicated, such as the tourniquet time, injection pressure, location, and the length of the procedure [6,9,13]. To our knowledge, no studies to date have included electromyography (EMG) to assess the involved nerves in an attempt to identify the location of the nerve dysfunction.

The purpose of this study was to determine variables that could increase the risk of a peripheral block complication and to identify patient demographic factors that may be able to predict an increased risk for a complication.

## Materials and methods

Institutional Review Board approval was obtained before the study began. We retrospectively reviewed charts of patients who underwent a foot and ankle operation between August 2013 and July 2018, as performed by the senior author. The review yielded 992 procedures across four surgical locations. The locations included three local hospitals and one outpatient surgery center. Of the 992 patients, 855 popliteal nerve blocks were administered. There were 468 popliteal nerve blocks performed in the outpatient surgery center. Of the 468, 257 (55%) were augmented with an adductor canal block. In the hospital setting, 387 popliteal nerve blocks were performed, with 255 (66%) receiving an additional saphenous nerve block. The use of the adductor canal or saphenous nerve block appeared to be due to provider preference. Postoperatively, the patients were analyzed for any nerve block-related complications. These were defined as sensory (paresthesia, numbness, tingling, or burning pain) or motor (weakness or paralysis) deficits along the distribution of the blocked nerve. Complications were noted by subjective complaints by the patients or on examination, such as objective motor weakness, sensory tenderness, or paresthesia. If a complication was present postoperatively, it was tracked to see if the complication resolved or if it caused long-term symptoms.

Anesthesiology data were also reviewed to evaluate anesthesia specific protocols for the administration of the blocks. All locations performed the block preoperatively and allowed the patient to be supine on the table. The nerve was located just proximal to the popliteal fossa, proximal to the bifurcation using an ultrasound. After locating the nerve, the area was prepped, and the nerve was reidentified with ultrasound and then confirmed with a nerve stimulator roughly 50% of the time. Next, 30 mL of 0.5% ropivacaine or 0.5% bupivacaine was injected in a single shot using a lateral approach. Epinephrine and corticosteroids were added to the injection based on the anesthesia provider’s preferences. The usage of epinephrine and corticosteroids could not be studied because the usage was not recorded in the anesthesia reports. The adductor canal or saphenous nerve was then injected with 5 mL to 10 mL of the same anesthetic used in the popliteal block.

Demographic factors of each patient were collected. These factors were age, sex, diabetic status, smoking, previous procedures, previously diagnosed neuropathies, surgical location, body mass index (BMI), race, and insurance provider. Additionally, the procedure that was being performed and the positioning on the table were noted. These factors were evaluated in patients who did and did not have a complication from the block to identify significant factors. Significance was determined by obtaining chi-square values for each data set. To analyze procedures, a binomial test was used to indicate if the proportion of complications for a procedure was lower than the expected rate of 0.5 (50%). This was chosen because of the null hypothesis that there is no difference in proportion of having a complication for each procedure. A regression analysis was also performed to identify the odds ratio for individual factors. All statistical analysis was performed using the SAS® software (SAS Institute Inc., Cary, North Carolina, USA).

## Results

The descriptive statistics of the study sample can be seen in Table 1. A total of 86 patients who received a block experienced a complication postoperatively (10.1%), of which 83 (96.5%) had sensory complications and 3 (3.5%) had motor complications. Of the 86 complication patients, 21 (24.4%) patients underwent EMG to evaluate for lingering nerve symptoms. EMGs were performed only on patients with severe neurologic symptoms with a duration of several months postoperatively, at the discretion of the senior author. Of these, 20 EMGs (95.2%) confirmed that the nerve symptoms were related to the nerve block given preoperatively. The EMGs demonstrated nerve symptoms that were proximal to the operative field at the popliteal fossa. The final EMG (4.8%) showed patterns consistent with fibromyalgia in the impression of the providing physician. Six (28.6%) patients who received an EMG had spontaneous resolution of symptoms, 10 (47.6%) patients were referred to a pain management specialist, and 5 patients (23.8%) had residual symptoms that the patient considered tolerable.

**Table 1:** Descriptive statistics of the study sample

Characteristic	N (%)
Age	
<40 years	237 (27.7)
40-65 years	469 (54.9)
>65 years	149 (17.4)
Sex	
Men	278 (32.5)
Women	577 (67.5)
Race/Ethnicity	
Non-Hispanic Whites	759 (88.8)
Other	96 (11.2)
Body Mass Index	
Underweight/Normal	194 (22.7)
Overweight	234 (27.4)
Obese	427 (49.9)
Insurance	
Medicare	162 (19.0)
Medicaid	21 (2.5)
Private	641 (75.0)
Other	31 (3.6)
Location	
Hospital 1	35 (4.1)
Hospital 2	335 (39.2)
Hospital 3	17 (2.0)
Outpatient Surgery Center	468 (54.7)
Smoking	
No	714 (83.5)
Yes	141 (16.5)
Diabetes	
Non-Diabetic	766 (89.6)
Diabetic	89 (10.4)
Previous Operations	
No	636 (74.4)
Yes	219 (25.6)
Previous Neuropathy	
No	803 (93.9)
Yes	52 (6.1)
Complication	
No	769 (89.9)
Yes	86 (10.1)
Complication Type	
Sensory	83 (96.5)
Motor	3 (3.5)

Out of the 86 patients, there were additionally 7 (8.1%) patients who were given an injection by a pain management specialist for their symptoms: 3 patients given peroneal injections and 4 given lumbar sympathetic injections. Two of the patients receiving peroneal and two receiving lumbar blocks were both referred for continued, scheduled blocks to relieve the symptoms. The remaining three patients had resolved symptoms after a single injection.

An additional three (3.5%) patients with a complication required radiofrequency ablation (RFA) of the injured nerve in order to relieve symptoms. After the ablation, all three patients reported resolution of the symptoms.

There were a remaining 55 (64.0%) patients of the 86 patients with complications who did not receive injections, EMGs, or RFAs. There were 43 (78.2%) patients who had spontaneous resolution of their symptoms. Additionally, five (9.0%) patients were referred to pain management, three (5.5%) were being followed by the senior author for continued symptoms, and four (7.3%) were referred to an outside provider for further work-up of their symptoms.

The final long-term complication rate of patients who did not have spontaneous resolution was 37 (4.3%) patients. For those patients with spontaneous resolution, symptoms took an average of 58.5 days to resolve. The patients with non-resolving symptoms were followed for a minimum of six months (nine patients; 24%) postoperatively with no resolution of symptoms. There were 28 (76%) patients who were followed for over one year postoperatively without resolution of symptoms. These previously discussed results of the 86 complication patients can be seen in Table 2.

**Table 2:** Outcome of complication patients EMG, electromyography

Outcome	n
Total Complication Patients	86
Total EMGs	21
Confirming EMGs	20
Symptoms Resolved	6
Pain Management	10
Residual Symptoms	5
Injections	7
Peroneal	3
Lumbar Sympathetic	4
Radiofrequency Ablation	3
Remaining Patients	
Spontaneous Resolution	43
Pain Management	5
Continued Personal Follow-Up	3
Outside Referral	4

The procedure comparison can be seen in Table 3. Table 3 contains the complete list of primary procedures performed on the study patients who had a resulting complication. The majority of the procedures within the table were significantly associated with a lower risk of complications. Those procedures that were not significantly associated with a complication were open reduction internal fixation (ORIF) of a talus fracture (P = 0.103), ORIF of a medial malleolus fracture (P = 0.257), tibiotalar joint arthrodesis (P = 0.096), ankle arthroscopy (P = 0.180), and incision and drainage procedures (P = 0.564). All procedures, except for primary Achilles tendon repairs and ORIF calcaneal fractures were performed in supine. There was 1 primary Achilles tendon repair performed in prone and 14 calcaneal fractures repaired laterally. Therefore, there was 1 procedure performed in prone, 14 procedures performed laterally, and 840 performed in supine. There was no statistical analysis performed for position of the patient on the table due to the limited number of prone and lateral procedures.

**Table 3:** Complication rates by procedure type ORIF, open reduction internal fixation

Procedure	Complication		P-Value
	No, n (%)	Yes n (%)	
Hallux Cheilectomy	11 (91.7)	1 (8.3)	0.004
ORIF Lateral Malleolus Ankle Fracture	109 (96.5)	4 (3.5)	<0.001
ORIF Trimalleolar Ankle Fracture	22 (91.7)	2 (8.3)	<0.001
ORIF Metatarsal Fracture	64 (88.9)	8 (11.1)	<0.001
Tarsometatarsal Joint Arthrodesis	107 (89.2)	13 (10.8)	<0.001
ORIF Calcaneal Fracture	11 (78.6)	3 (21.4)	0.034
ORIF Pilon Fracture	8 (88.9)	1 (11.1)	0.020
Modified Brostrom	149 (81.4) 8	34 (18.6)	<0.001
ORIF Talus Fracture	5 (83.3)	1 (16.7)	0.103
Calcaneal Osteotomy	41 (91.1)	4 (8.9)	<0.001
Talonavicular Joint Arthrodesis	21 (91.3)	2 (8.7)	<0.001
Hardware Deep Removal	19 (90.5)	2 (9.5)	<0.001
ORIF Ankle Syndesmosis	25 (92.6)	2 (7.4)	<0.001
ORIF Medial Malleolus Fracture	5 (71.4)	2 (28.6)	0.257
Gastrocnemius Recession	55 (94.8)	3 (5.2)	<0.001
Tibiotalar Joint Arthrodesis	7 (77.8)	2 (22.2)	0.096
Ankle Scope with Extensive Debridement	4 (80)	1 (20)	0.180
Incision and Drainage	2 (66.7)	1 (33.3)	0.564

In the demographic analysis, the significant factors associated with a complication were age (P = 0.0061), BMI (P = 0.0031), location (P = 0.0016), and smoking status (P = 0.0026). Factors that were not significantly associated with complications were sex, diabetes status, previous procedures requiring a block, previously diagnosed neuropathies, race, and insurance provider. A regression analysis was performed to determine the odds ratio for individual factors. Those with significantly higher odds ratios were between 40 and 65 years of age, had a normal or underweight BMI, underwent surgery at an outpatient surgery center, and were current smokers. The demographic analysis can be seen in Tables 4, 5. Table 4 demonstrates the characteristic breakdown of the study population by complication status, and Table 5 demonstrates the results of the regression analysis.

**Table 4:** Characteristics of the study sample by complication status

Characteristics	Complications		P-Value
	No, n (%)	Yes, n (%)	
Age			0.0061
<40 years	214 (90.3)	23 (9.7)	
40-65 years	411 (87.6)	58 (12.4)	
>65 years	144 (96.6)	5 (3.4)	
Sex			0.0533
Men	258 (92.8)	20 (7.2)	
Women	511 (88.6)	66 (11.4)	
Race/Ethnicity			0.8132
Non-Hispanic Whites	682 (89.9)	77 (10.1)	
Other	87 (90.6)	9 (9.4)	
Body Mass Index			0.0031
Underweight/Normal	162 (83.5)	32 (16.5)	
Overweight	216 (92.3)	18 (7.7)	
Obese	391 (91.6)	36 (8.4)	
Insurance			0.2245
Medicare	152 (93.8)	10 (6.2)	
Medicaid	20 (95.2)	1 (4.8)	
Private	570 (88.9)	71 (11.1)	
Other	27 (87.1)	4 (12.9)	
Location			0.0016
Hospital 1	32 (91.4)	3 (8.6)	
Hospital 2	317 (94.6)	18 (5.4)	
Hospital 3	16 (94.1)	1 (5.9)	
Outpatient Surgery Center	404 (86.3)	64 (13.7)	
Smoking			0.0026
No	652 (91.3)	62 (8.7)	
Yes	117 (83.0)	24 (17.0)	
Diabetes			0.9858
Non-Diabetic	689 (90.0)	77 (10.1)	
Diabetic	80 (89.9)	9 (10.1)	
Previous Operations			0.8001
No	573 (90.1)	63 (9.9)	
Yes	196 (89.5)	23 (10.5)	
Previous Neuropathy			0.3999
No	724 (90.2)	79 (9.8)	
Yes	45 (86.5)	7 (13.5)	

**Table 5:** Regression analysis results demonstrating odds ratios

Characteristic	Adjusted OR (95% CI)	P-Value
Age		
<40 years	3.37 (0.93, 12.15)	0.063
40-65 years	4.77 (1.42, 16.03)	0.011
>65 years	[Reference]	
Sex		
Men	[Reference]	
Women	1.72 (0.99, 2.99)	0.056
Race/Ethnicity		
Non-Hispanic Whites	[Reference]	
Other	0.91 (0.43, 1.95)	0.809
Body Mass Index		
Underweight/Normal	[Reference]	
Overweight	0.39 (0.21, 0.74)	0.004
Obese	0.47 (0.26, 0.82)	0.009
Insurance		
Medicare	[Reference]	
Medicaid	0.42 (0.05, 4.02)	0.455
Private	0.63 ( 0.24, 1.66)	0.351
Other	0.81 (0.19, 3.42)	0.777
Location		
Hospital 1	0.66 (0.19, 2.33)	0.514
Hospital 2	0.35 (0.19, 0.63)	0.000
Hospital 3	0.40 (0.05, 3.223)	0.387
Outpatient Surgery Center	[Reference]	
Smoking		
No	[Reference]	
Yes	2.23 (1.29, 3.87)	0.004
Diabetes		
Non-Diabetic	[Reference]	
Diabetic	1.49 (0.67, 3.32)	0.334
Previous Operations		
No	[Reference]	
Yes	1.16 (0.68, 1.99)	0.580
Previous Neuropathy		
No	[Reference]	
Yes	1.41 (0.58, 3.45)	0.448

## Discussion

Multiple studies have examined the complication rate associated with peripheral nerve blocks [7-16]. The complication rate ranges from 0%, as reported by a study conducted by Provenzano et al. [16] with 467 patients, to 1.6%, reported by a study conducted by Klein et al. [9] with 2,382 blocks. Additionally, some studies examined short-term complication rates. Kahn et al. [14] showed a 7.2% short-term rate and Park et al. [15] showed an 11% short-term rate.

This study showed a short-term rate of 10.1% (86 patients) on 855 blocks done, which is consistent with the range shown in the literature, although on the higher side. However, the long-term complication rate was 4.3% (37 patients), which is higher than what has been published in the current literature. There are seven patients included in the long-term rate who had symptoms resolved by an injection or RFA, and those excluded would drop the complication rate to 3.5%.

In addition to the complication rate, the study was focused on identifying factors that may be able to predict a complication in a patient. The study by Anderson et al. [13] identified age and tourniquet pressure as significant factors resulting in complications. The average age associated with postoperative symptoms was 47.3 years and that without symptoms was 50.2 years [13]. They found that those with postoperative complications had a tourniquet pressure of 6 mm Hg lower than those without complications (303.4 and 309.4 mmHg, respectively) [13]. However, they did not believe that these were significant factors clinically. Gartke et al. [17] did not find significance with tourniquet time, tourniquet placement, or patient age. Since this study evaluated age by groups instead of averages, it is difficult to correlate the results with the study by Anderson et al. [13], and it contradicts the findings by Gartke et al. [17]. Additionally, the senior author consistently places a very proximal thigh tourniquet and uses a tourniquet pressure of 300 mm Hg for all procedures. Since the author uses the same tourniquet pressure for each procedure, the pressure was not tracked during this study. The tourniquet time was not tracked as well due to the short nature of the procedures being performed.

The procedures noted to have a predicted incidence of complications are infrequently performed procedures. Isolated talus and medial malleolus fractures are rarely encountered, ankle arthroscopy is routinely performed with other procedures, and the senior author has moved away from tibiotalar joint fusion in favor of ankle arthroplasty when appropriate.

 This study found several significant factors, including age, BMI, location, and smoking status. The regression analysis allowed for a specific patient population to be identified with an increased odds ratio. Those with the highest incidence of having a complication related to the peripheral block were patients who were 40-65 years of age, had normal or underweight BMI, underwent surgery at an outpatient surgery center, and were current smokers. We believe that this is useful information in discussing the risks and benefits with a patient preoperatively. We can identify patients meeting some of these criteria and come to an informed decision. An open discussion can decrease the morbidity for patients postoperatively. This discussion can decrease morbidity by decreasing the amount of long-term complications in high-risk patients by identifying the risks associated with peripheral nerve blocks.

It is not clearly understood what causes these persistent neurological complications. In a review by Jeng et al. [6], they stated that intrafascicular injections along with high injection pressures can result in neuronal injury and that a long-beveled needle can cause mechanical injury to the nerves when compared to a short-beveled needle. The needle bevel length used for patients in this study was unable to be determined. The anesthesia records for patients stated the brand, gauge, and length of the overall needle but did not indicate the bevel length. Kapur et al. [18] performed a canine study that examined injection pressures and the neurologic outcomes. They noted that 8 dogs with increased injection pressures had lingering neurologic symptoms when compared to 12 dogs with lower injection pressures [18]. Injection pressures were not measured but could be measured in future studies. In addition, the senior author began obtaining EMGs to evaluate the nerve pain late in the data collection period to try and identify the location of the nerve problem. What it has shown is that the nerve symptoms can be attributed to the nerve block near the popliteal fossa and no other factors. It would have been helpful to collect preoperative EMGs as well to demonstrate the change in findings from a patient’s baseline. It could have provided further details on the impact of the nerve block. A proposed factor in some anesthesia studies was mechanical damage caused by the surgeons during the operation [8,11]. The EMGs obtained in this study confirmed that the nerve complications are proximal to the operative field near the popliteal fossa. Therefore, if nerves are injured during the operative procedure, they are not reproducing the symptoms seen on the EMGs. Having collected the EMGs from the beginning of the study would have been helpful; however, having a 95% positive finding with those sampled would lead one to believe that it would be consistent if done throughout the study.

When relating these studies to the demographic factors, it is uncertain why the higher risk population in this study had higher complication rates. We are unsure why those between 40 and 65 years of age had significantly higher complication rates. We were most surprised by those with underweight or normal BMI having the highest odds ratio. We have two theories that describe the increased risk of this population. The first is that a thinner person may have an increased chance of mechanical damage to the nerve with the needle. Nielsen et al. [19] looked at nerve blocks in overweight and obese individuals and concluded that the problem with administering blocks is difficulty in locating appropriate landmarks. Although the landmarks are easier to visualize, there is less fatty tissue to serve as padding around the nerves. The decreased padding may increase the susceptibility of a thin person to needle mechanical damage [19]. The second is that there is less fat surrounding the nerve to absorb any excess anesthetic, although ropivacaine typically used is less lipophilic than bupivacaine [20]. We speculate that the outpatient surgery center had more complications because of a procedural difference of the anesthesiologists. The procedure outlined in this study was gathered by discussions with providing anesthesiologists and procedural records. This is a commonality among providers, but there may still be differences from provider to provider. An example is that at the local hospitals, a saphenous nerve block was performed 66% of the time to supplement the popliteal block and an adductor canal block was performed 55% of the time to supplement the popliteal block at the outpatient surgery center. Our study showed that smoking is a significant predictor of postoperative complications, similar to the finding by Gartke et al. [17]. The mechanism by which smoking causes complications may be related to the impact that smoking has by decreasing cortical excitability [21]. Smoking has been shown to decrease peripheral nerve function as well. Agrawal et al. [22] showed that 16.7% of stable chronic obstructive pulmonary disease (COPD) patients may have subclinical peripheral neuropathy. The patients in the study demonstrated altered EMG findings from the control group [22]. In a review by Gupta and Agarwal [23], they conclude that studies show up to one-third of COPD patients have clinical signs of peripheral neuropathy and two-thirds of patients have EMG abnormalities. Of the neuropathy symptoms, the most prevalent among patients were sensory symptoms [23]. These studies show that smoking patients may have an underlying subclinical neuropathy that becomes clinical with the use of a peripheral nerve block.

This study has some strengths that allow for generalizability and decreased bias. This is a multicenter study that contains blocks performed by many different anesthesiologists. The study also accounts for a wide variety of procedures that can be performed by an orthopedic foot and ankle specialist. The breadth of location, anesthesiologists, and procedures allow for generalizability of the data. Although there are strengths in the study, there are some limitations to the study. With it being a multicenter study, having many anesthesiologists who performed blocks on the study patients can be seen as a limitation. Although we outline the common procedure between the four sites, there is still a technique variation for each provider. Additionally, it is a retrospective study that carries an inherent bias. The senior author recognized the problem of nerve complications and wished to study his trends. In this study, 24.4% of the patients underwent an EMG. Increasing this number can further show an association between the blocks and the complications. Another limitation of the study is the tourniquet time and pressure not being tracked. In future studies, these factors can be tracked and analyzed for complications.

## Conclusions

In conclusion, our study identifies that age between 40 and 65 years, normal or underweight BMI, surgery at an outpatient surgery center, and current smoking could be significant factors in predicting a peripheral nerve block complication in a patient undergoing foot and ankle surgery. Along with these factors, we identified that the short-term complication rate is similar to previous studies, although our long-term rate was higher. We were also able to demonstrate in a subset of patients that the nerve symptoms could be localized to the area around the popliteal fossa based upon EMG findings. We believe that further research can examine the injection pressures in patients, especially the higher risk group from this study, to see if that could be a predictor of complications postoperatively.
